# Case report: Atypical anaphylactic reaction to Patent Blue V dye during breast cancer surgery

**DOI:** 10.3389/fonc.2022.979393

**Published:** 2022-09-16

**Authors:** Stanka Misir Šitum, Iva Korečić Zrinjščak, Maja Pečvarac, Andrea Šoštar, Ana Žaja, Tea Tot

**Affiliations:** Department of Anesthesiology, University Hospital for Tumors, University Hospital Center Sestre Milosrdnice, Zagreb, Croatia

**Keywords:** breast cancer, patent blue V dye, anaphylactic reaction, anesthesia, case report

## Abstract

The most common causes of perioperative hypersensitive reactions are neuromuscular blocking drugs, latex, and antibiotics, although there are other more emerging causative agents. Allergic reactions to Patent Blue V (PBV) dye have been reported. Most of them are mild and presented with blue coloration of cutaneous plaque. The PBV dye is widely used in the identification of sentinel lymph nodes in patients with breast cancer and other malignancies. Here, we present a case of 33-year-old patient with carcinoma of the breast proposed for sentinel lymph node and skin-sparing mastectomy with severe, life-threatening anaphylaxis which occurred immediately after PBV dye was injected, with cardiopulmonal resuscitation and prolonged refractory hypotension. The patient was without previous exposure to PBV and signs of skin rash, erythema, or bronchospasm, making the diagnosis and management of such cases challenging. Skin tests were performed on all drugs used in premedication and induction of anesthesia and PBV showed positive at IDT of 1:10. Physicians must always think of possible adverse reaction to PBV and for the potential risk of anaphylactic reaction immediately after the dye is injected, during anesthesia and other procedures.

## Introduction

Although the most common causes of perioperative anaphylactic reactions are neuromuscular blocking drugs (70%), latex (10%), and antibiotics, there are other more emerging causative agents (1, 2). The Patent Blue V (PBV) dye is widely used in the identification of sentinel lymph nodes in patients with breast cancer and other malignancies (3–5). PBV dye can provoke allergic reaction of varying degrees of severity (6, 7). Most often these are mild reactions in the form of urticaria or erythema without hemodynamic and respiratory disorders, but sometimes can cause severe life-threating anaphylactic reaction (6, 8). These reactions occur immediately or a few minutes to several hours after the injection of the dye (9). The most serious allergic reaction is anaphylactic shock with circulatory failure (7, 8, 10, 11).

We present an unusual case of severe anaphylaxis to PBV dye with atypical clinical features in a young woman with carcinoma of the right breast.

## Case presentation

A 33-year-old patient with carcinoma of the right breast proposed for sentinel lymph node and skin sparing mastectomy, followed by reconstruction with prosthesis, had a history of iodine, cefalexine and trastuzumab allergies. The patient had one previous surgery without complications and no prior medical contact with PBV dye.

After anesthetic induction and endotracheal intubation, the surgeon applied 1 mL of Patent Blue V Sodium Guerbet 2.5%, diluted to 2 mL with normal saline, subcutaneously into the retromamilar peritumoral region of the breast for the identification of the sentinel lymph node and massaged the application site. Immediately after injection of the PBV dye, the patient developed volume refractory hypotension of 50/30 mmHg, bradycardia with 30–45 beats per minute, desaturation, and skin goosebumps, without signs of skin rash, erythema, urticaria, edema, or bronchospasm. These symptoms required prompt treatment and we started with intravenous administration of diluted epinephrine, first fractionated total of 1 mg, then continuous infusion of 0.8 mg/h. The patient also received boluses of norepinefrine 0.01 mg/ml, 250 mg of hydrocortisone, 100 mg of ranitidine, 20 mg of chloropyramine, 2 mg of athropine, 3000 mL of crystalloids, and 500 mL of colloids. For 20 min after application of the PBV dye, the patient remained in severe hypotension and bradycardia, and ventricular fibrillation rhythm occurred. Following immediate cardiopulmonal resuscitation with cardiac massage for 1 min and defibrillation using 200 J, we managed the conversion in the sinus rhythm at around 120 beats per minute. The patient received treatment with 300 mg of amiodarone, 60 mL of bicarbonates, and electrolyte correction, and then an arterial cannula and urinary catheter were placed with invasive blood pressure measurement. Surgical intervention was not performed, and the patient was transported to the intensive care unit (ICU) intubated, hypotensive 75/45 mmHg, on continuous infusion of epinephrine 0.5 mg/h, and normal skin with mild cervical edema. After 1 h of admission to ICU, the patient was extubated, promptly awake, without any neurocognitive deficit. During the first 8 h in the ICU, the patient was very hemodynamically unstable, with tachycardia up to 150 beats per minute and hypotension with systolic pressure of 70–90 mmHg, on continuous infusion of epinephrine 0.5 mg/h and boluses of norepinephrine 0.02 mg iv. After that, the patient demanded support with continuous infusion of epinephrine 0.05–0,3 mg/h for a total of 44 h. Within the first 24 h, the patient received 7800 mL of intravenous fluid, and spontaneous diuresis of greenish blue urine was 4700 ml, which stayed greenish blue for 36 h. The patient was discharged from the ICU at Day 3 in good condition. No tryptase assay was done.

Seven weeks later, at the allergy department, skin and challenge tests were performed on all drugs used in premedication and induction of anesthesia (midazolam, etomidat, fentanyl, rocuronium, ceftriaxon), and results were negative. These medications were allowed for use, and the risk of anaphylactic reaction was not higher than other members of the population. We also tested Patent Blue V sodium Guerbet 2.5% using the following protocol: skin prick test (SPT) 1:10, 1:1, and intradermal skin test (IDT) 1:1000, 1: 100, 1:10. The Patent Blue V sodium Guerbet 2,5% was positive at IDT of 1:10.

After 3 months, the patient was reoperated without the use of blue dye and with all the other drugs used in the first surgery, without complications. Instead of sentinel lymph node, the surgeon performed an axillary dissection.

## Discussion

Our case is an example of atypical life-threatening anaphylactic reaction that presented with severe and prolonged hypotension refractory to epinephrine, without signs of skin rash, erythema, urticaria, edema, or bronchospasm.

The most common triggers of drug-induced allergic reactions during surgery are neuromuscular relaxants, antibiotics, anesthetic induction drugs, opioids, colloids, NSAIR, topical antiseptics, and latex (12). All drugs used in premedication and induction of anesthesia were excluded as triggers of anaphylaxis with negative skin and challenge tests. The NSAIR drugs were not given. Colloids were used for the treatment of anaphylactic reaction. Skin prick allergy tests to latex and topical antiseptics were not performed and they could be potential triggers. However, that was unlikely in this case, because the latex gloves and the same topical antiseptics were used in the second surgery after 3 months without any complication. The presented symptoms left no doubt that it was an anaphylactic reaction, although serial tryptase measurement was not performed to confirm the diagnosis of anaphylaxis. Investigation of PBV dye associated allergy sensitivity to triptase, skin prick, and intradermal test was 54%, 80% and 100% respectively (9). From all this it remains to be assumed that is was an allergic reaction to PBV dye.

This patient never had contact with medical blue dye. In most reported cases there was a lack of previous exposure to PBV dye (2, 5, 7, 11, 13). Mechanisms underlying allergic reactions to blue dyes have not been fully elucidated. Most likely, an IgE-mediated activation of mast cell causes this allergic response (8, 9). An IgE-dependent response must, of course, be preceded by exposure to blue dyes. PBV dye, belonging to the family of dyes from triphenylmethane, also known as E131, disulfide blue, or acid blue 3, is extensively used for commercial purposes such as in colorants in everyday objects; as a food additive; in the textile, paper, and agriculture industries; and in cosmetic and medical products; and this can explain prior sensitization (5, 8, 11). There is evidence that about 2,7% of the population would be allergic to blue dye (7).

The incidence of anaphylactic reaction of any severity associated with PBV dye is about 0.1%–2.8% (3, 12, 13). The median time to anaphylaxis was 20–30 min with a range of 0–90 min (14). Severe and potentially life-threating anaphylactic shock is rare, with an incidence of 0.06% (9, 12).

Reactions to PBV dye usually consist of skin lesions (blue coloration of the cutaneous plaque) and hypotension, but respiratory symptoms are uncommon. Reactions tend to occur 15–30 min after the dye is injected and onset is faster for more serious reactions (15). Immediate anaphylaxis is uncommon (9, 10). In our case, anaphylactic shock occurred immediately after the PBV dye is injected, with refractory hypotension, bradycardia, and skin goosebumps, but the blue coloration of the skin was not present. In a review, Barthelmes et al. reported two patients who experienced a severe reaction within 1 min of the administration of PBV dye and three patients within 5 min (9). Telgenkamp et al. reported anaphylactic shock with cardiac arrest immediately after the injection of PBV dye (8). There is no data on the proportion of patients lacking skin blue coloration and there is currently no validated explanation for this (13).

Although anaphylaxis usually presents as an acute episode, the mast cell can release mediators and support anaphylaxis for hours (16). PBV dye is excreted within 24–48 h, mainly in the urine, which is intensively stained, but also in the bile (3, 16). This patient was hypotensive and required vasoactive support for 44 h, and we assumed that these reactions are due to the long-life PBV dye and slow release of the PBV dye from the parenchymal tissue that could trigger reaction until the blue dye was completely eliminated from the body.

Anaphylactic shock after PBV dye injection remains a serious adverse event and warrants awareness (8). Immediate action with cardiac resuscitation, adrenergic support, mast cell-stabilizing medications, and corticosteroids can stabilize the patient. Vasopressor response may be obtained with a starting dose of the drug or may require prolonged drug infusion. Cardiovascular system depression may be prolonged and require cardiovascular support and admission to the intensive care unit (6). In high-risk patients, preventive anti-allergic medications should be applied before blue dye injection (8). Fortunately, there are no cases of death described in the literature due to an allergic reaction to the PBV dye (7, 12).

All surgical patients should give consent for adverse reactions to PBV dye preoperatively (1). Physicians must remain constantly vigilant with patients undergoing procedures using the blue dye (16). Particularly during anesthesia, one must always think of possible adverse reactions to PBV dye and for the potential risk of anaphylactic reaction, immediately after the dye is injected, even in the absence of blue coloration of the cutaneous plaque. Alertness and early intervention play important roles, and prompt treatment is needed for those patients with anaphylaxis.

## Data availability statement

The original contributions presented in the study are included in the article/supplementary material. Further inquiries can be directed to the corresponding author.

## Ethics statement

Written informed consent was obtained from the individual(s) for the publication of any potentially identifiable images or data included in this article.

## Author contributions

SMŠ, IKZ performed the anesthesia and ICU treatment. AŽ, MP, AŠ and TT provided supervision and participated in the literature review and in drafting the manuscript. All authors contributed to the article and approved the submitted version.

## Conflict of interest

The authors declare that the research was conducted in the absence of any commercial or financial relationships that could be construed as a potential conflict of interest.

## Publisher’s note

All claims expressed in this article are solely those of the authors and do not necessarily represent those of their affiliated organizations, or those of the publisher, the editors and the reviewers. Any product that may be evaluated in this article, or claim that may be made by its manufacturer, is not guaranteed or endorsed by the publisher.
